# Modulating hESC-derived cardiomyocyte and endothelial cell function with triple-helical peptides for heart tissue engineering

**DOI:** 10.1016/j.biomaterials.2020.120612

**Published:** 2021-02

**Authors:** Maria Colzani, Jean-Daniel Malcor, Emma J. Hunter, Semih Bayraktar, Murray Polkinghorne, Thomas Krieg, Ruth Cameron, Serena Best, Richard W. Farndale, Sanjay Sinha

**Affiliations:** a Department of Medicine and Wellcome – MRC Cambridge Stem Cell Institute, University of Cambridge, Cambridge, UK; b Department of Biochemistry, University of Cambridge, UK; c Department of Medicine, University of Cambridge, Cambridge, UK; d Department of Materials Science and Metallurgy, University of Cambridge, UK

**Keywords:** Cardiac tissue engineering, Collagen biomaterials, Triple-helical peptides, Pluripotent stem cells, Regenerative medicine

## Abstract

In this study, we investigated the role of cardiomyocyte (CM) and endothelial cell (EC) specific interactions with collagen in the assembly of an operational myocardium *in vitro*. Engineered cardiac patches represent valuable tools for myocardial repair following infarction and are generally constituted of a suitable biomaterial populated by CMs and supportive cell types. Among those, ECs are required for tissue vascularization and positively modulate CM function. To direct the function of human embryonic stem cell (hESC)-derived CM and EC seeded on biomaterials, we replicated cell-collagen interactions, which regulate cellular behaviour in the native myocardium, using triple-helical peptides (THPs) that are ligands for collagen-binding proteins. THPs enhanced proliferation and activity of CMs and ECs separately and in co-culture, drove CM maturation and enabled coordinated cellular contraction on collagen films. These results highlight the importance of collagen interactions on cellular response and establish THP-functionalized biomaterials as novel tools to produce engineered cardiac tissues.

## Graphical abstract

**Figure:**
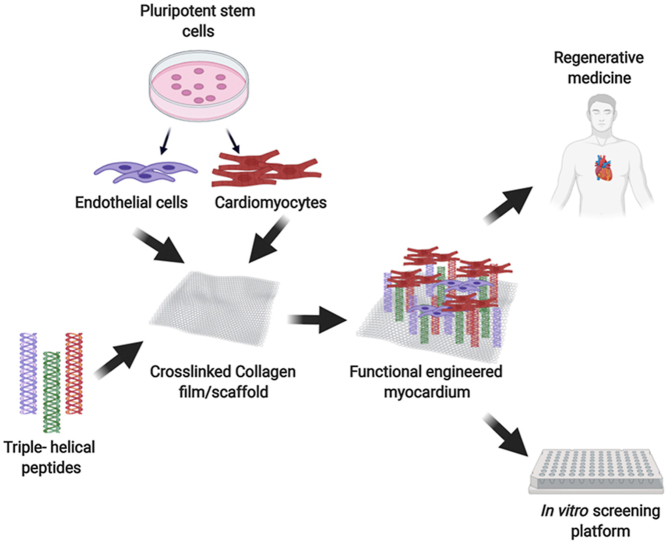

## 1 Introduction

Recovery of cardiomyocyte (CM) loss represents the main challenge in patients suffering from ischemic heart failure. Current therapies fail to address this loss by means other than cardiac transplantation. Pluripotent stem cell-derived CMs have emerged as new tools to treat infarcted hearts [1,2]. CMs can be injected and successfully engraft, align and couple with the host myocardium and improve cardiac function [3]. However, the vast majority of cells die upon injection, in part due to early washout or anoikis. Delivering CM in the form of a suitable cardiac patch may circumvent these limitations. Long term patch survival and integration with the host tissue vasculature are key issues to address. Incorporating endothelial cells (ECs) in the patch therefore represents a promising solution. Several groups have developed engineered heart tissues [[4], [5], [6], [7], [8]]. However, despite recent advances, significant issues still need to be addressed such as the optimal cell composition, matrix components and mechanical properties needed to recapitulate the myocardium. In particular, cell interaction with the extra-cellular matrix (ECM) especially collagen, the main constituent of the ECM, has been generally overlooked despite its potential to direct cell fate and function [[9], [10], [11]].

Here, we propose to investigate the role of CM- and EC-specific interactions with collagen in the assembly of engineered tissues. Collagen is widely used to produce biomaterial scaffolds [12,13]. Collagen crosslinking naturally occurs to provide collagen fibrils with biomechanical capabilities and adequate resistance. In tissue engineering, this crosslinking is often substituted by 1-ethyl-3-(3-dimethylamino-propyl)carbodiimide (EDC) and N-hydroxysuccinimide (NHS) treatment [14] leading to stiff materials with long term structural integrity. However, EDC/NHS treatment recruits and consumes aspartate, glutamate and lysine residues from the collagen sequence that would otherwise be involved in cellular interaction [15,16]. EDC/NHS crosslinked collagen biomaterials thus reproduce the physical characteristic of the myocardium but are biologically inert to many cell types.

In this study, cell-collagen interactions in crosslinked collagen materials were re-instated by grafting specific triple-helical peptides (THPs). THPs are composed of three peptide strands, each constituted of a series of GXX’ triplets. They adopt a triple helix conformation with a one amino acid stagger mimicking the natural structure of collagen. In particular, THPs containing the GFOGER or GLOGEN motif are ligands for collagen-binding integrins α1β1, α2β1, α10β1 and α11β1 [17,18]. GPRGQOGVMGFO and its derivative GPRGQOGVNleGFO (referred to as VWFIII_Nle_ in the literature) are sequences targeting the discoidin domain receptors (DDRs) 1 [19] and 2 [20], the von Willebrand Factor (VWF) [21] and the Secreted Protein Acidic and Rich in Cysteine (SPARC) [22,23]. THPs containing GFOGER or GPRGQOGVNleGFO sequences restored and enhanced non-cardiac cell function on 2D collagen film models in our previous proof-of-concept work [24,25].

We have thus designed an engineered tissue composed of collagen films functionalized with THPs and populated with human embryonic stem cell (hESC)-derived CMs and ECs separately or in co-culture. We identified the correct set of THPs that enabled optimal cell attachment, survival, proliferation and function. Overall, investigating cellular response to THPs gave us insight into the influence of collagen-binding protein on cell function and could represent a new tool to generate next generation engineered myocardial patches. Our methodology can be extended to other relevant cell types, such as epicardial cells, cardiac fibroblasts or smooth muscle cells [26]. THP-functionalized crosslinked collagen films could provide a convenient engineered heart tissue for the *in vitro* study of cardiac pathologies. Furthermore, this technology could be transposed to three-dimensional scaffolds for the manufacturing of patches for damaged heart repair.

## 2 Results

### 2.1 Collagen film functionalization with THPs

**Fig. 1:**
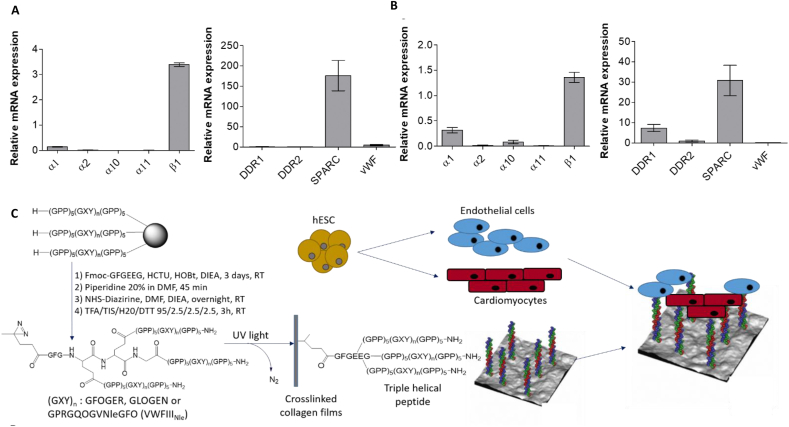
Crosslinked collagen films are functionalized with THPs targeting collagen-binding proteins and seeded with endothelial cells and cardiomyocytes. A) and B) Relative mRNA expression of genes coding for collagen-binding integrins subunits (α1, α2, α10, α11 and β1), DDR1, DDR2, SPARC and vWF was examined in endothelial cells (A) and cardiomyocytes (B). mRNA from three independent cell batches of hESC-derived ECs and CMs ready for seeding upon collagen films was isolated, purified and transcribed into complementary cDNA. cDNA was amplified and quantified by qPCR (compared to a house-keeping gene GAPDH for integrin subunits and HPRT1 for DDR1, DDR2, SPARC and vWF); graphs show mean ± s.e.m. Integrin α subunits mRNA expression ranking was α1 > α2 > α10 and α11 for ECs and α1 > α10 > α2 and α11 for CMs. VWFIII_Nle_-binding proteins mRNA expression ranking was SPARC ≫ vWF > DDR1 and DDR2 for ECs, and SPARC > DDR1 > DDR2 and vWF for CMs. C) Synthetic pathway of collagen film functionalization with THPs. Peptides that contain the active sequences GFOGER (targeting preferentially α2β1 and α11β1 integrins), GLOGEN (targeting preferentially α1β1 and α10β1 integrins) or GPRGQOGVNleGFO (targeting DDR1, DDR2, vWF, SPARC) are assembled on solid support by Fmoc/tBu strategy, end-stapled using the GFGEEG hexapeptide, reacted with NHS-Diazirine and removed from the support to yield photoreactive THPs. Photoreactive THPs were incubated on crosslinked collagen films and covalently attached to the collagen backbone upon UV treatment. Non-crosslinked films, crosslinked films with no peptide or films functionalized with THPs are then populated with hESC-derived ECs and CMs.

To predict possible THP interactions with ECs and CMs, the expression of mRNA for collagen-binding proteins were assessed by RT-qPCR (Fig. 1, A-B). β1 integrin subunit was expressed at high levels in both cell types. ECs express predominantly the α1 integrin subunit (0.14 ± 0.02), relatively small amounts for α2 (0.02 ± 0.006) and no α10 and α11. CMs also expressed α1 the most (0.32 ± 0.05), followed by α10 (0.087 ± 0.03), α2 (0.02 ± 0.002) and α11 (0.01 ± 0.001). Integrin expression at protein level was also assessed by flow cytometry. Mean fluorescence intensity (MFI) quantification showed a similar trend to the mRNA data. However, it revealed that the differences in protein expression between collagen-binding integrins are not as pronounced, with a lower but non-negligible expression of integrin α2 at the cell surface both of endothelial cells and cardiomyocytes (Supporting information 1). GFOGER binds preferentially, but not exclusively, to α2β1 and α11β1 and GLOGEN binds preferentially to α1β1 and α10β1 (Supporting information 2) [17,18]. Taking into consideration both the protein and mRNA expression data, we expect GLOGEN to contribute more efficiently than GFOGER to cell affinity for the substrate.

Among the non-integrin collagen receptors, DDR1, DDR2, vWF and SPARC bind to the VWFIII_Nle_ motif [[20], [21], [22]]. Both cell types expressed considerably higher levels of SPARC (176.10 ± 37.39 for ECs and 30.78 ± 7.50 for CMs) relative to other VWFIII_Nle_-binding proteins. vWF (5.08 ± 1.73) was also detected in ECs and mRNA for DDR1 was detected in CMs (7.37 ± 1.81). DDR2 (0.48 ± 0.26 for ECs and 0.99 ± 0.47 for CMs), DDR1 in ECs (0.97 ± 0.38) and vWF in CMs (0.004 ± 0.01) were expressed in lesser amounts (Fig. 1, A-B). We therefore predicted that VWFIII_Nle_ will mainly interact with SPARC secreted by ECs and CMs. Nevertheless, VWFIII_Nle_ may also influence cell response by interacting with vWF secreted by ECs and through DDR1 in CMs. Overall these results prompted us to generate collagen films functionalized with GFOGER, GLOGEN, VWFIII_Nle_, GFOGER + VWFIII_Nle_, or GLOGEN + VWFIII_Nle_ as described previously [24,25](Fig. 1, C).

### 2.2 ESC-derived EC behaviour on collagen films

**Fig. 2:**
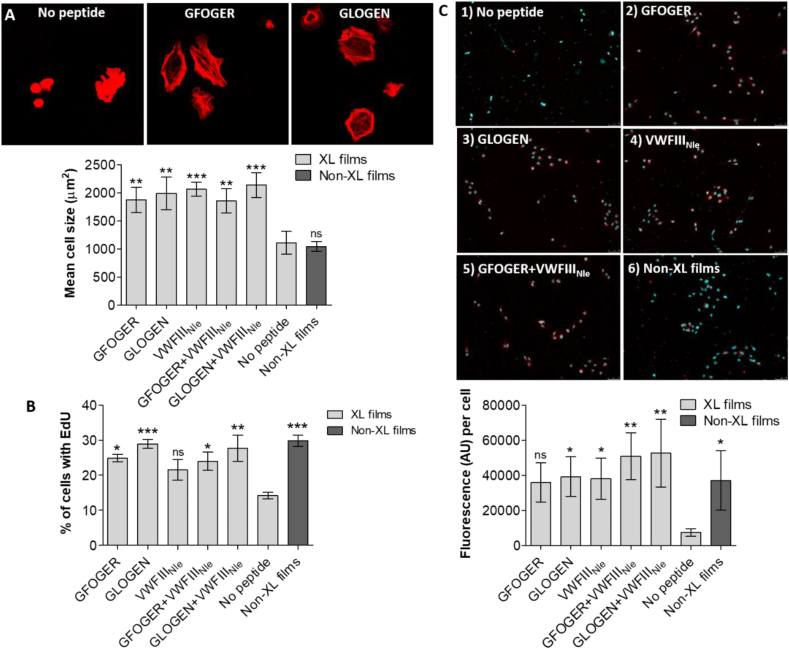
**Endothelial cell cultures on crosslinked collagen films without peptide, functionalized with GFOGER, with GLOGEN, with VWFIII**_**Nle**_**and with GFOGER + VWFIII**_**Nle**_**, or non-crosslinked films.** A) EC spreading. After 45 min at 37 °C with 5% CO_2_, hESC-derived ECs were fixed and stained with Rhodamine-Phalloidin. Representative fields of view for GFOGER and GLOGEN are shown. Mean cell area was calculated from 10 randomly selected fields of view per conditions in three independent repeats (n = 3) and plotted as mean ± s.e.m. THP functionalization consistently increased mean cell surface area (one-way ANOVA, p < 0.001). Columns were compared to crosslinked films with no peptides using Dunnett's post-hoc test. B) EC proliferation. hESC-derived ECs were cultured for 24 h at 37 °C with 5% CO_2_. EdU was introduced in the media and cells were left for 2h at 37 °C with 5% CO_2_. Cells were fixed, nuclei were stained with Hoechst 33342 and EdU was stained with Alexa Fluor-488. The ratio of cells with EdU-Alexa488 over the total number of cells was calculated from 10 randomly selected fields of view per conditions in three independent repeats (n = 3) and plotted as mean ± s.e.m. THP functionalization consistently increased the percentage of proliferating ECs after 24h (one-way ANOVA, p < 0.01). Columns were compared to crosslinked films with no peptides using Dunnett's post-hoc test. C) EC LDL uptake. hESC-derived ECs were cultured in cell culture media containing Ac-LDL-Dil for 24 h at 37 °C with 5% CO_2_, fixed and stained with Hoechst 33342. Representative fields of view are shown. Mean Dil fluorescence intensity per cell was measured in 10 randomly selected fields of view per conditions in four independent repeats (n = 4) and plotted as mean ± s.e.m. THP functionalization consistently increased the uptake of acetylated LDL by ECs (one-way ANOVA, p < 0.01). Columns were compared to crosslinked films with no peptides using Dunnett's post-hoc test.

ECs were seeded on crosslinked collagen films (noted XL in figures) without peptides or functionalized with GFOGER, GLOGEN or VWFIII_Nle_ as well as on non-crosslinked films (noted Non-XL in figures). Surprisingly, without peptides, ECs adhere to crosslinked films in a magnesium-dependent manner, indicating integrin dependence (Supporting information 3). While EDC/NHS treatment affects integrin recognition, binding motifs may not be fully ablated and were sufficient to support initial cell adhesion. Significant differences, however, were observed in cell spreading (Supporting information 4). Peptides grafted on crosslinked films increased cell size in all conditions (1875 ± 226.1 μm,^2^ p < 0.01 for GFOGER, 1991 ± 292.3 μm,^2^ p < 0.01 for GLOGEN, 2065 ± 124.9 μm,^2^ p < 0.001 for VWFIII_Nle_, 1855 ± 216.4 μm,^2^ p < 0.01 for GFOGER + VWFIII_Nle_ and 2139 ± 222.4 μm,^2^ p < 0.001 for GLOGEN + VWFIII_Nle_) when compared to the crosslinked collagen films without peptide (1110 ± 204 μm^2^) (Fig. 2, A). Unexpectedly, ECs seeded on non-crosslinked films displayed a similar morphology as on crosslinked films (1046 ± 87.39 μm^2^).

Next, EC proliferation 24h after seeding was investigated. 14.24 ± 0.93% of ECs were EdU positive on crosslinked films without peptide. This percentage rose to 24.88 ± 1.11% with GFOGER (p < 0.05), 28.96 ± 1.32% with GLOGEN (p < 0.001), and 21.49 ± 2.95% with VWFIII_Nle_ (not significant). Combining VWFIII_Nle_ with GFOGER (23.99 ± 2.60%, p < 0.05) and GLOGEN (27.68 ± 3.72%, p < 0.01) also led to an increase in proliferation compared to non-functionalized crosslinked films, but not compared to crosslinked films grafted with THPs individually (Fig. 2, B; supporting information 5).

EC function was investigated via uptake of acetylated low-density lipoprotein (Ac-LDL) (Fig. 2, C). Ac-LDL-Dil was detected around the cell nucleus and the levels Ac-LDL-Dil fluorescence per cell rose from 7395 ± 2248 AU on 100% crosslinked films to 35841 ± 11241 AU (not significant), 39161 ± 11326 AU (p < 0.05) and 38064 ± 11670 AU (p < 0.05) with GFOGER, GLOGEN and VWFIII_Nle_ respectively. The combination of VWFIII_Nle_ and integrin-binding peptides resulted in a more significant increase in Ac-LDL-Dil uptake (50942 ± 13433 AU, p < 0.01 with GFOGER and 52726 ± 19337 AU, p < 0.01 with GLOGEN). On non-crosslinked collagen films Ac-LDL-Dil was detected (37125 ± 17003 AU per cell), but rarely accumulated in the cytoplasm. Taken together these results indicated that GFOGER, GLOGEN and VWFIII_Nle_ facilitate the cellular response of ECs.

### 2.3 ESC-derived CMs behaviour on collagen films

**Fig. 3:**
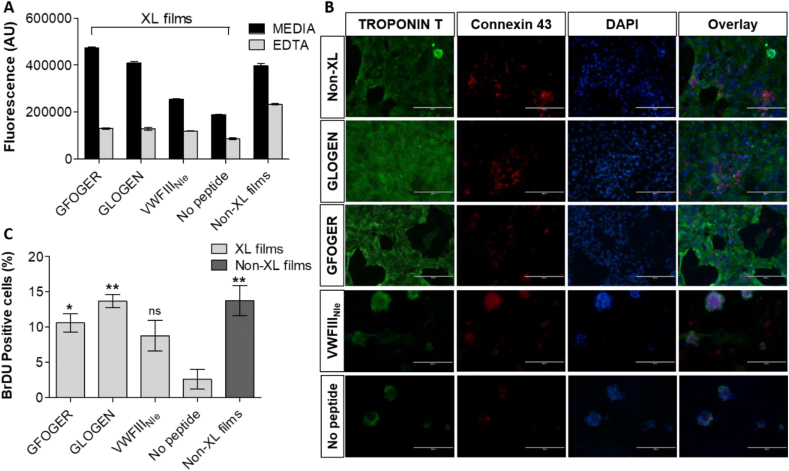
**Cardiomyocyte cultures on crosslinked collagen films without peptide, functionalized with GFOGER, with GLOGEN and with VWFIII**_**Nle**_**, or non-crosslinked films.** A) CM viability. After 5 days at 37 °C with 5% CO_2_, PrestoBlue® with and without 5 mM EDTA was added to cell culture media, fluorescence at 560 nm was measured in three independent repeats (n = 3) and plotted as mean ± s.e.m. GFOGER and GLOGEN significantly restored CM viability abolished by EDC/NHS treatment after 5 days (one-way ANOVA, p < 0.001). Columns were compared to crosslinked films with no peptides using Dunnett's post-hoc test. B) CM immunocytochemistry. hESC-derived CMs were cultured for 5 days at 37 °C with 5% CO_2_ and stained with Troponin T-Alexa Fluor-488, Connexin-43-Alexa Fluor-568 and DAPI. Representative fields of view are shown. Panels from left to right show Troponin T, Connexin-43 and DAPI staining, and merge. CM cultured on crosslinked films without peptide or with VWFIII_Nle_ formed clumps, whereas CMs cultured on crosslinked films with GFOGER or GLOGEN, or on non-crosslinked films formed monolayers with intercellular punctate gap junctions. Scale bar 100 μm. C) CM proliferation. hESC-derived CMs were cultured for 3 days at 37 °C with 5% CO_2_. BrdU was introduced in the media and cells were left for 12h further at 37 °C with 5% CO_2_. Cells were fixed and nuclei were stained with DAPI and internalized BrdU with Alexa-568. The ratio of cell nuclei with BrdU over the total number of nuclei was calculated from 5 randomly selected fields of view per conditions in three independent repeats (n = 3) and plotted as mean ± s.e.m. GFOGER and GLOGEN significantly increased the percentage of proliferating CMs after 4 days (one-way ANOVA, p < 0.01). Columns were compared to crosslinked films with no peptides using Dunnett's post-hoc test.

CMs were cultured on functionalized collagen films and their viability was assessed by PrestoBlue® at day 5 (Fig. 3, A). Viability was comparable between non-crosslinked films and crosslinked films with GFOGER or with GLOGEN (396197 ± 10993.65, 472769.7 ± 4505.828 and 408811 ± 6063.209 AU respectively). Significantly lower values were obtained on crosslinked films without peptides or with VWFIII_Nle_ (189906 ± 1564.068 and 254070.7 ± 2735.761 respectively) (Fig. 3. Therefore, CMs viability is significantly reduced by the EDC/NHS treatment and can be fully restored by grafting THP ligands for the collagen-binding integrins, whereas VWFIII_Nle_-mediated signalling only has a limited effect. The addition of EDTA resulted in a dramatic drop in fluorescence on all crosslinked films (between 85960.66 ± 2161.564 and 130479.7 ± 1785.082 AU and 233853.7 ± 1640.333 AU on non-crosslinked films). Fluorescence was not completely ablated in the presence of EDTA indicating a potential role for non-integrin collagen receptors in CMs adhesion. However, the remaining cells were lost during the subsequent staining procedure suggesting only a loose attachment via non-integrin mechanisms.

Troponin-T staining showed that CMs formed a monolayer and Connexin-43 staining revealed punctate intercellular communications on films non-crosslinked or crosslinked and functionalized with GFOGER or GLOGEN. In contrast, cells clustered into clumps on crosslinked films with no peptide or with VWFIII_Nle_, with diffuse Connexin-43 staining and some Troponin-T negative cells, implying possible CM dedifferentiation (Fig. 3, B).

Further, CM proliferation 4 days after seeding was investigated (Supporting information 6). Collagen film crosslinking almost entirely ablated cell proliferation (from 13.79 ± 2.136% on non-crosslinked films to 2.606 ± 1.418% on crosslinked films). THP ligands re-established the proportion of proliferating cells back to 8.767 ± 2.171% with VWFIII_Nle_ (not significant), 10.62 ± 1.295% with GFOGER (p < 0.05) and 13.70 ± 0.9436% with GLOGEN (p < 0.01). GLOGEN was more efficient than GFOGER in restoring cell proliferation, which in turn was more efficient than VWFIII_Nle_ (Fig. 3, C).

**Fig. 4:**
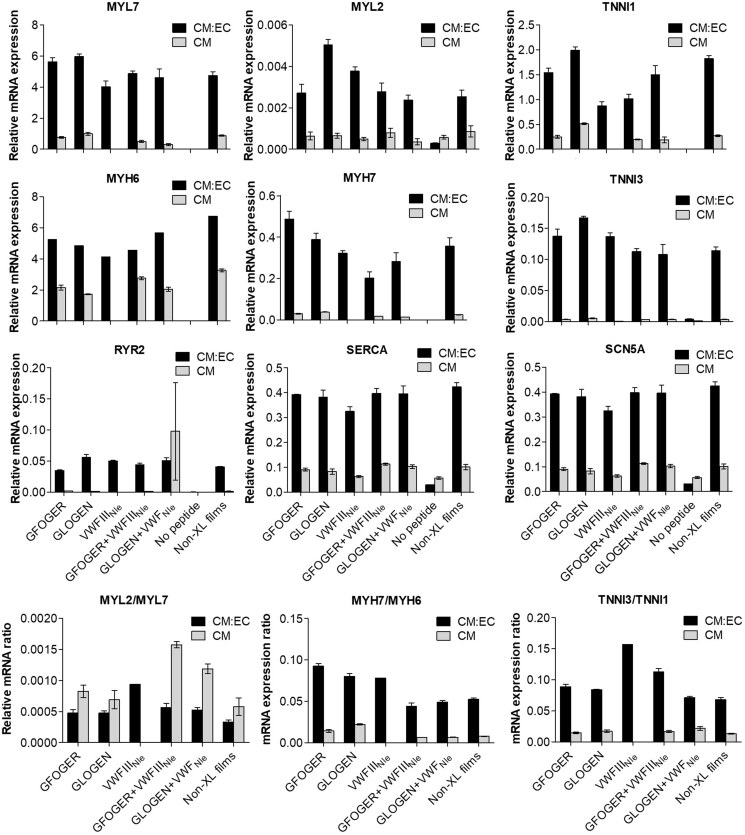
**Relative mRNA expression of cardiac genes in CMs cultured alone or with ECs.** mRNA from three independent batches (n = 3) of CM only or CM:EC cultures after 5 days was isolated, purified and transcribed into complementary cDNA. cDNA was amplified and quantified by qPCR compared to a GAPDH house-keeping gene (graphs show mean ± s.e.m.). MYL7, MYH6, MYH7, TNNI1, TNNI3, RYR2 and SCN5a were upregulated with GFOGER, GLOGEN, GFOGER + VWFIII_Nle_ and GLOGEN + VWFIII_Nle_ for CM only cultures (one-way ANOVA, p < 0.0001). All genes were strongly upregulated with all sets of THPs for EC:CM co-cultures (one-way ANOVA, p < 0.0001). Adult isoform/foetal isoform gene expression with GFOGER, GLOGEN, VWFIII_Nle_, GFOGER + VWFIII_Nle_, GLOGEN + VWFIII_Nle_ and non-crosslinked films was calculated for MYL2/MYL7, MYH7/MYH6 and TNNI3/TNNI1. CM only cultures resulted in higher MYL2/MYL7, while CM:EC co-cultures resulted in higher MYH7/MYH6 and TNNI3/TNNI1 ratios (one-way ANOVA, p < 0.0001). Overall, THPs in CM:EC co-cultures guided most advanced CM maturation.

We also examined expression of genes associated with sarcomeric assembly (Fig. 4). In addition to the conditions above, CMs were also seeded on films functionalized with GFOGER + VWFIII_Nle_ and GLOGEN + VWFIII_Nle_. Except MYL2, the expression of which remained consistent across all conditions, all genes were more abundantly expressed on crosslinked films with GFOGER and GLOGEN compared to without peptides or with VWFIII_Nle_ (479-, 234-, 947-, 211- and 2.5-fold increases in average for MYL7, MYH6, MYH7, TNNI1 and TNNI3 respectively). MYH7/MYH6, MYL2/MYL7 and TNNI3/TNNI1 ratios were also calculated as indicators of CM maturation. Since sarcomeric genes (besides MYL2) were virtually absent in CMs seeded on crosslinked films without peptides or with VWFIII_NIe_, ratios could not be calculated meaningfully for these conditions. All other conditions yielded similar ratios, except for MYL2/MYL7 that was higher with GFOGER + VWFIII_Nle_ and GLOGEN + VWFIII_Nle_. Additionally, we probed for calcium handling (RYR2 and SERCA) and ion-channel (SCN5a) genes. Compared to crosslinked films with no peptide, all three were upregulated (20-fold for RYR2, 1.7-fold for SERCA and 2.5-fold for SCN5a in average) in the presence of integrin-binding THPs. VWFIII_Nle_, however, did not contribute. Overall, CM gene expression demonstrate more advanced CM maturation in the presence of integrin-binding THPs.

**Fig. 5:**
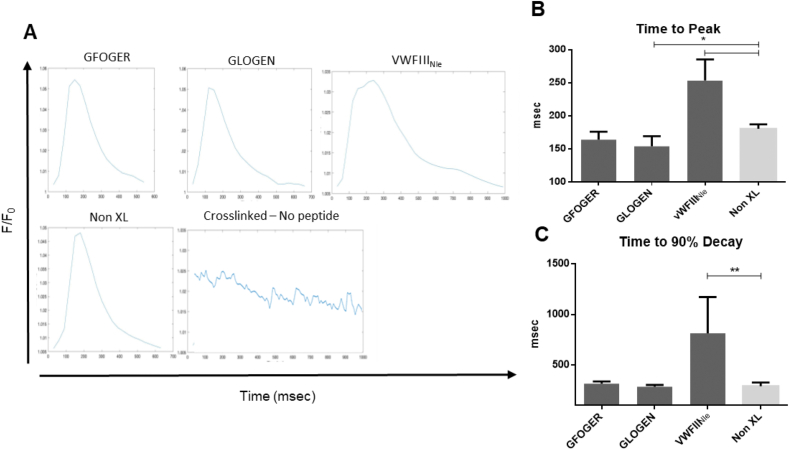
**CM calcium release and CM contraction.** hESC-derived CMs were cultured for 5 days at 37 °C with 5% CO_2_, loaded with Fluo-4AM and electrically paced at 1.5 Hz. **A**) Transient calcium influx was assessed by measuring Fluo-4AM fluorescence intensity above background (F/F_0_) as a function of time in four independent repeats (n = 4). CMs seeded on non-crosslinked films and crosslinked films functionalized with GFOGER or GLOGEN showed regular and synchronised contractions. Time to peak (**B**) and Time to 90% decay (**C**) are plotted as mean ± s.e.m. Columns were compared to non-crosslinked films using Dunnett's post-hoc test. GLOGEN significantly improved both time to peak and time to 90% decay while VWFIII_NIe_ performed significantly worse (p < 0.05, Student's t-test). Crosslinked films with no peptides are not shown as no defined peak to analyse were identifiable.

Calcium handling using Fluo-4 AM on cells paced at 1.5 Hz was then analysed (Fig. 5, A, Supporting videos). Time to peak (TTP; Fig. 5, B) and time to 90% decay (T90; Fig. 5, C) were measured to reflect the rapidity and synchronicity of CM response to electrical stimuli. Coordinated calcium release could not be observed on crosslinked films without peptides, defined peaks were poorly recorded, and the resulting parameters could not be calculated. In contrast, CMs contracted regularly (TTP 180.625 ± 5.694, T90 291 ± 30.759) on untreated collagen films. On crosslinked films, VWFIII_Nle_, even if not always following the pacing, caused calcium release at regular spaced intervals. However, both TTP and T90 were significantly higher than on non-crosslinked films (253.375 ± 27.733 and 815.125 ± 310.078 respectively, p < 0.05). In contrast, GFOGER and GLOGEN, both prompted regular and synchronised beating. Moreover, both TTP and T90 were comparable to the untreated control films with a small but significant decrease in the TTP with GLOGEN (TTP 164.125 ± 10.454 and 153.875 ± 13.263, p < 0.05; T90 315.625 ± 18.403 and 284.875 ± 16.226 respectively). Overall, integrin-binding THPs had a compelling positive impact on CM phenotype and behaviour, at least comparable to non-crosslinked collagen, while VWFIII_Nle_ had only a limited and non-significant effect.

**Fig. 6:**
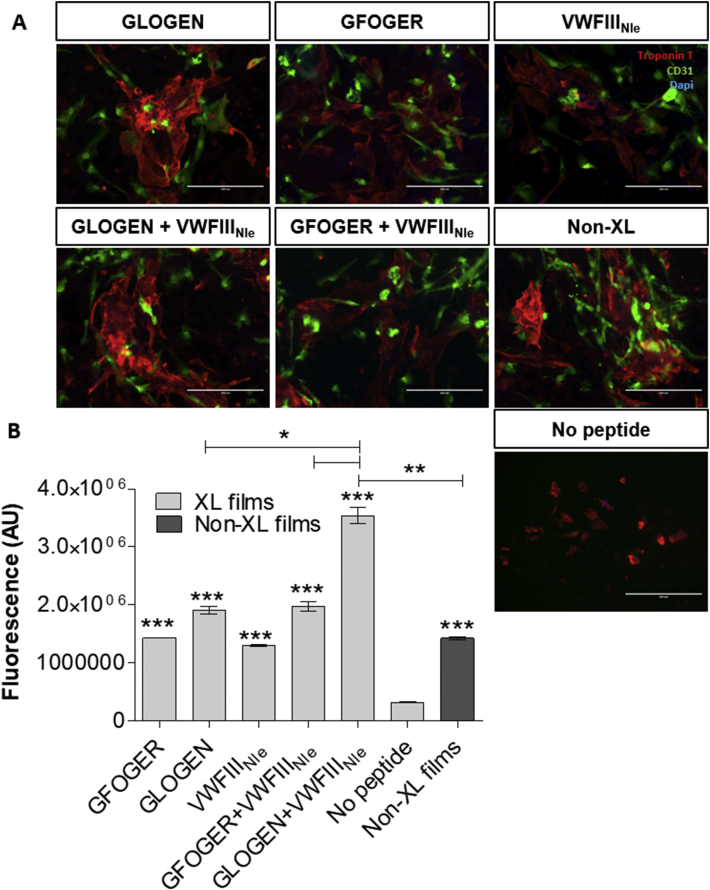
**Endothelial cells and cardiomyocytes co-cultures on crosslinked collagen films without peptide, functionalized with GFOGER, with GLOGEN, with VWFIII**_**Nle**_**, with GFOGER + VWFIII**_**Nle**_**, with GLOGEN + VWFIII**_**Nle**_**, or non-crosslinked films.** A) CM:EC immunocytochemistry. hESC-derived ECs and CMs were cultured alongside for 5 days at 37 °C with 5% CO_2_ and stained with Troponin T (CM specific marker), CD31 (EC specific marker) and DAPI. Representative fields of view of merged fluorescence are shown. Scale bar 100 μm B) CM:EC viability. After 5 days at 37 °C with 5% CO_2_, PrestoBlue® was added to cell culture media, fluorescence at 560 nm was measured in three independent repeats (n = 3) and plotted as mean ± s.e.m. All sets of THPs significantly restored CM viability abolished by EDC/NHS treatment after 5 days (one-way ANOVA, p < 0.001). Columns were compared to crosslinked films with no peptides using Dunnett's post-hoc test. GLOGEN + VWFIII_Nle_ resulted in significantly higher cell viability than all other conditions (Student's t-test).

The effect of THPs in EC:CM co-cultures was investigated. Cells were seeded at a 10:1 ratio of CM:EC and cultured for 5 days. We first verified that both cell types survived and maintained their phenotype by CD31 and Troponin-T staining (Fig. 6, A). Cell growth over 5 days and viability was assessed using PrestoBlue® (Fig. 6, B). Again, the overall cell metabolic activity was dramatically affected by EDC/NHS crosslinking (dropping from 1.427 × 10^6^ ± 27285 to 322667 ± 9838 AU) and could be restored by grafting GFOGER (1.43 × 10^6^ ± 5774, p < 0.001), GLOGEN (1.91 × 10^6^ ± 69282, p < 0.001), but also VWFIII_Nle_ (1.303 × 10^6^ ± 17638, p < 0.001). We hypothesized that although VWFIII_Nle_ does not affect CMs, it promotes EC activity and in turn has an effect in CM:EC co-culture. Moreover, GFOGER + VWFIII_Nle_ (1.975 × 10^6^ ± 80978, p < 0.001) and GLOGEN + VWFIII_Nle_ (3.543 × 10^6^ ± 145307, p < 0.001) had a stronger impact than each peptide grafted individually (p < 0.05). GLOGEN + VWFIII_Nle_ yielded best results, with significantly higher viability than on native collagen films (p < 0.01).

Expression of CM maturation markers was re-evaluated in CMs cultured with ECs (Fig. 4) and compared to expression in CM only cultures. CM gene expression was significantly increased on crosslinked films functionalized with THPs or on non-crosslinked films, compared to crosslinked films without peptides (1.15 × 10^4^-fold for MYL7, 10-fold for MYL2, 7254-fold for MYH6, 5208-fold for MYH7, 1567-fold for TNNI1, 30-fold for TNNI3, 140-fold for RYR2, 13-fold for SERCA and 10-fold for SCN5a in average). Unlike in CM only cultures, VWFIII_Nle_ strongly upregulated gene expression, as importantly as GFOGER and GLOGEN. MYH7/MYH6, MYL2/MYL7 and TNNI3/TNNI1 ratios were calculated for all sets of THPs and followed the same pattern as on CM only cultures. VWFIII_Nle_ individually also augmented MYH7/MYH6, MYL2/MYL7 and TNNI3/TNNI1 ratios compared to CMs seeded on non-crosslinked films. Compared to CM only cultures, MYH7/MYH6 and TNNI3/TNNI1 but not MYL2/MYL7, values were higher, implying a more advanced CM maturation. Of note, in the co-cultures cardiac gene expression was consistently increased compared to the CM only cultures, in line with what has been previously reported [27].

**Fig. 7:**
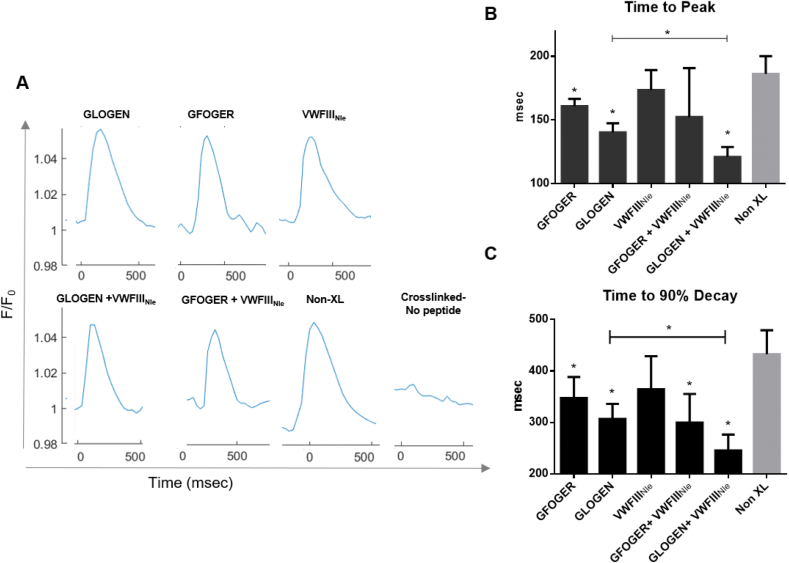
**CM:EC calcium release and contraction.** hESC-derived CMs and ECs were cultured for 5 days at 37 °C with 5% CO_2_, loaded with Fluo-4AM and electrically paced at 1.5 Hz. **A**) Transient calcium influx was assessed by measuring Fluo-4AM fluorescence intensity above background (F/F_0_) as a function of time in three independent repeats (n = 3). Time to peak (**B**) and Time to 90% decay (**C**) are plotted as mean ± s.e.m. Columns were compared to non-crosslinked films using Dunnett's post-hoc test. In the presence of endothelial cells, GLOGEN, GFOGER and GLOGEN + VWFIII_Nle_ significantly improved both time to peak and time to 90% decay while VWFIII_NIe_ performed similarly to the control (p < 0.05, Student's t-test). Crosslinked films with no peptides are not shown as no defined peak to analyse were identifiable. GLOGEN + VWFIII_Nle_ showed significant improvement also compared to GLOGEN alone (p < 0.05, Student's t-test).

Finally, the contractile ability of CMs in EC:CM co-cultures was evaluated (Fig. 7). Crosslinked films with no peptide still did not support CM beating. Non-crosslinked films supported coordinated beating but performed worse than with CM only cultures (TTP 186.229 ± 12.036, T90 432.71 ± 40.499). Contrary to CMs alone, in the presence of VWFIII_Nle_ cells contracted similarly as on the non-crosslinked controls (TTP 173.656 ± 13.486, T90 364.685 ± 55.596). A significant decreased in both TTP and T90 was observed with GFOGER (TTP 160.988 ± 4.908, T90 347.599 ± 35.263; p < 0.05), followed by GLOGEN (TTP 140.238 ± 6.251, T90 306.800 ± 25.428, p < 0.05) and GLOGEN + VWFIII_Nle_ (TTP 121.003 ± 6.820, T90 245.253 ± 27.024 p < 0.01). With GFOGER + VWFIII_Nle_ however, only T90 was significantly shorter (TTP 152.378 ± 33.342, T90 299.553 ± 48.448 p < 0.05). GLOGEN associated with VWFIII_Nle_ was the best condition, with more rapid impulses, compared notably to both GLOGEN alone (p < 0.05) and native collagen. To further investigate the possible mechanism behind the synergistic effect observed on GLOGEN + VWFIII_Nle_ films, focal adhesion kinase (FAK) phosphorylation (p-FAK), was analysed by flow cytometry in endothelial cells/cardiomyocytes co-culture. FAK is of relevance has it has been linked to cardiac contractility [28]. Supporting information 7 shows that in cardiomyocytes p-FAK levels are significantly higher on GLOGEN + VWFIII_Nle_ films compared to Non-XL and GLOGEN only functionalized surfaces, confirming a similar trend to what was observed in Fig. 7 with the calcium transient analysis.

## 3 Discussion

Several models of engineered myocardium are being developed by considering different factors such as mechanical constraints [29] and electric stimulation [30]. Here, we instead aimed to employ specific and controlled interactions with the ECM, and in particular with collagen, to modulate ECs and CMs adhesion, proliferation and function. The most prevalent ECM proteins in the myocardium are collagen types I and III, in contact with CMs, and type IV, in contact with the basal lamina of ECs. Collagen is required for heart tissue organisation, resistance to mechanical workloads and plays a major role in EC organization [31] and CM contraction [32]. However, all the pathways involving collagen-binding proteins have yet to be determined, thus hindering the control of hESC-derived EC or CM fate on biomaterials.

To elucidate these mechanisms and to fabricate active biomaterials, we utilized EDC/NHS-treated collagen films. EDC/NHS crosslinking is required to obtain stiff and stable materials, but drastically reduces recognition for crucial collagen-binding proteins, rendering the substrate biologically inert.

The influence of EDC/NHS treatment on collagen film mechanical properties has previously been investigated by our group. The crosslinking method used in this work (with a 5:2:1 molar ratio of EDC:NHS:carboxylic acid moieties in collagen, referred to as 100% in our previous studies) raised the tensile Young's modulus of 2D collagen films from 5.7 MPa to 31 MPa [33]. This crosslinking is also necessary to ensure structural integrity of 3D collagen porous scaffolds, increasing their compressive modulus from 1.2 kPa to 6.2 kPa [15]. Since mechanical constraints play a central role in heart function and myocardial remodelling post-infarct, adequate mechanical properties obtained through EDC/NHS treatment are especially important for cardiac tissue engineering. A heart patch should provide a structural template for cell attachment and tissue formation that ideally matches the mechanical properties of the myocardium [34]. While a rigid but inelastic patch placed on the heart will impede contraction, a soft biomaterial will not be sufficiently resistant to withstand changes in strains in the native myocardium [35]. Moreover, the heart patch should not biodegrade before cardiac repair has been initiated. EDC/NHS crosslinking of collagen biomaterials thus constitutes a useful tool to achieve stable and resistant cardiac patches that can be used in regenerative medicine.

Overall this methodology enabled us to tailor CM and EC interactions with collagen films and direct cell fate by grafting the correct set of THP ligands [36]. Despite their potential, THPs remain rarely used in tissue engineering [24,25,[37], [38], [39]]. In this study we aimed to determine the influence of collagen-binding protein recognition on hESC-derived CM and ECs cell response.

First, we explored THPs' influence on hESC-derived ECs. Cell spreading was significantly increased with GFOGER, GLOGEN and VWFIII_Nle_ compared to the control with no peptide. EC proliferation was enhanced with the addition of all THPs and consistent with previous results. THPs also improved Ac-LDL uptake in EC cytoplasm. Surprisingly, on non-crosslinked films, where integrin binding sites are intact ECs did not spread and only few cells contained Ac-LDL. Non-crosslinked films may not be stable in cell culture media and might not provide a stable surface for ECs. Alternatively, collagen films, made from type I collagen, may not be the optimal surface for the ECs, naturally in contact with collagen type IV. Grafting collagen type I films with THPs saturates the surface with binding sites for integrins and VWFIII_Nle_-binding proteins, thus supplanting the need for collagen type IV binding sites. Overall, these results validate the positive impact of THP ligands on EC function. Due to VWFIII_Nle_ affinity for several collagen-binding proteins, it is difficult to distinguish through which pathway this peptide affects ECs. Nevertheless, mRNA expression in ECs suggests that SPARC would be the main partner for VWFIII_Nle_ [40,41].

CMs establish strong intercellular interactions that lead to spontaneous organized contraction. This was achieved by day 5 on non-crosslinked films or crosslinked films with integrin-binding peptides but not without peptides or with VWFIII_Nle_ where CMs formed clumps and were less viable. Proliferation studies corroborated further this observation. With clinical applications in mind, we aimed to produce substrates on which CM can proliferate to populate a biomaterial at high density highlighting the importance of functionalizing collagen-based constructs with the integrin-binding THPs.

hESC-derived CMs are relatively immature, possess rudimentary sarcoplasmic reticulum and express fetal form of key sarcomeric proteins [42]. We thus analysed expression of key genes associated with mature CMs. Compared to crosslinked films all genes were upregulated when interacting with GFOGER and GLOGEN, but not VWFIII_Nle_. Integrin-binding THPs thus stimulated gene expression changes associated with CM maturation, reaching equivalent or higher levels than on native collagen. CM on crosslinked film underwent sparse and random contraction. Contrastingly, VWFIII_Nle_ prompted a regular calcium release but with significantly higher TTP and T90 compared to untreated collagen films. GFOGER or GLOGEN, however, restored synchronised beating to the pattern obtained on non-crosslinked films, highlighting once again the importance of the integrin-mediated interactions with collagen for the maturation, organization, and function of CMs.

Unlike ECs, CMs are naturally in contact with collagen type I (along with type III) and consequently displayed viability, proliferation, maturation and coordinated beating on non-crosslinked films. These features are lost following EDC/NHS treatment, but were fully restored with the addition of integrin binding ligands, GFOGER or GLOGEN. VWFIII_Nle_, however, only has a limited and often non-significant effect, despite CMs expressing genes coding for DDR1 and SPARC. Overall, both for ECs and CMs, GLOGEN had a slightly more important effect that GFOGER (both alone and in combination with VWFIII_Nle_). Both cell lines express dramatically more α1β1 than other collagen-binding integrins. We attributed this difference in cellular response to GLOGEN higher affinity for α1β1 than GFOGER, thus sparking greater repercussions.

Interactions between different cell types are pivotal for the assembly, organization and physiological role of tissues. We therefore performed co-cultures of CMs and ECs, as vascularization is a vital element for maintaining artificial tissues. As expected, the addition of THP ligands led to a significant improvement of cell growth. Interestingly, VWFIII_Nle_ alone also ameliorated cell viability as much as GFOGER. Moreover, cell viability was dramatically improved with GLOGEN in combination with VWFIII_Nle_.

Addition of THP ligands, including VWFIII_Nle_ alone, upregulated all the genes that were examined with identical patterns as in CM-only cultures. However, this upregulation was strikingly exacerbated in CM:EC co-cultures. This trend, confirmed by similarly greater MYH7/MYH6 and TNNI3/TNNI1 ratios (but not MYL2/MYL7, higher with CMs only), indicates an advanced CM maturation when seeded alongside ECs. Finally, paced CM contraction was re-evaluated in co-cultures with ECs. Again, crosslinked films did not support any coordinated calcium release. Remarkably, in CM:EC co-culture VWFIII_Nle_ alone significantly improved calcium handling to levels equal to the non-crosslinked films. GFOGER and GFOGER with VWFIII_Nle_ lead to a decrease in both TTP and T90, although the addition of VWFIII_Nle_ did not lead to any additional beneficial effect. In comparison, a synergistic effect was observed with GLOGEN associated with VWFIII_Nle_, which provided the best outcome with significantly faster calcium handling both compared to the untreated collagen films control and to GLOGEN alone. This synergistic effect was not observed with GFOGER leading to the hypothesis that GLOGEN exerts a slightly more pronounced effect due to its higher affinity for α1β1. This was confirmed in FAK phosphorylation experiments, a key event in the integrin signalling pathway that has been previously linked to cardiac contractility [28]. When GLOGEN is used in combination with VWFIII_Nle_, FAK phosphorylation is increased compared to the non-crosslinked films or GLOGEN-functionalized films, while this is not the case for GFOGER (Supporting information 7). These results reinforce both our observations that GLOGEN has a stronger effect than GFOGER, and that the association with VWFIII_Nle_ enhances the effect of GLOGEN. Of note, CMs with ECs on non-crosslinked films exhibited less acute impulses than with CMs alone. This may be explained by the lack of EC affinity for non-crosslinked films, leading to a detrimental impact on CM function. Overall, these results illustrate how a factor impacting on one cell type has repercussions on the global cell population.

Our results demonstrate that CM and EC behaviour can be adjusted by functionalizing otherwise inert cell matrices with THP ligands for collagen-binding proteins. THP-functionalized crosslinked collagen biomaterials can thus be a novel and powerful tool for the design of an engineered myocardium with features that can be adapted by using different THPs. From a fundamental perspective, our study has contributed to assert the importance of collagen interactions with ECs and CMs on cell function. Having mechanical properties that match that of the human heart, functionalized collagen films can also constitute easy-to-use 2D platforms for *in vitro* assays and tissue engineering research. Our efforts are currently directed towards applying this technology to three-dimensional collagen-based scaffolds for the fabrication of patches for cardiac tissue repair that can operate as closely as possible to the native organ.

## 4 Materials and methods

### 4.1 General procedure for peptide synthesis

9-Fluorenylmethoxycarbonyl (Fmoc) protected amino acids and N, N-Dimethylformamide (DMF) were supplied by AGTC Bioproducts (Hessle, UK). Fmoc-protected 6-Aminohexanoic acid (Ahx) was supplied by Merck (Darmstadt, Germany). All other amino acids and reagents were purchased from Sigma-Aldrich (Gillingham, UK). Peptides were synthesized as previously described [24,25]. Briefly, (GPP)_5_GFOGER(GPP)_5_, (GPP)_5_GLOGEN(GPP)_5_, Ahx-(GPP)_5_GPRGQOGVNleGFO(GPP)_5_ and GPC(GPP)_10_GPC (GPP10) were synthesized as C-terminal amides on Rink amide aminomethyl Tantagel resin (0.526 g, loading 0.19 mmol.g-1, RAPP Polymere) by Fmoc/tert-butyl solid phase strategy. Peptide GFGEEG was synthesized as C-terminal acid on Fmoc-Gly-Wang (0.127 g, 0.79 mmol g^−1^, Novabiochem). N-terminal end-stapling of three peptide strands of (GPP)_5_GFOGER(GPP)_5_ (GPP),_5_GLOGEN(GPP)_5_ and Ahx-(GPP)_5_GPRGQOGVNleGFO(GPP)_5_ (5.10^–5^ mol) was performed on solid support by reaction with Fmoc-GFEEG-OH (4.5 mg, 5.6.10^–6^ mol) followed by Fmoc deprotection in DMF with 20% piperidine for 2 × 15 min. DIEA (5.7 μl, 3.3.10^–5^ mol) and NHS-Diazirine (5.63 mg, 2.5.10^–5^ mol, Life Technologies) were dissolved in DMF and added to resin beads (8.3.10^–6^ mol) in the dark overnight to give Diazirine-coupled end-stapled (GPP)_5_GFOGER(GPP)_5_ (denoted GFOGER), (GPP)_5_GLOGEN(GPP)_5_ (denoted GLOGEN) and Ahx-(GPP)_5_GPRGQOGVNleGFO(GPP)_5_ (denoted VWFIII_Nle_). Cleavage from the resin was performed in a mixture of trifluoroacetic acid (TFA)/triisopropylsilane (TIS)/H2O 95/2.5/2.5 v/v/v for 2h. The cleavage solution was concentrated and precipitated in 20 ml of cold diethyl ether. The white precipitate was filtered, washed with 10 ml of cold diethyl ether, dissolved in a H2O/acetonitrile (ACN) 95/5 v/v (0.1% TFA) and freeze dried. The crude product was purified by preparative reverse-phase high performance liquid chromatography (RP-HPLC) on a PerkinElmer LC200 system equipped with a 10 μm Eurospher II 100-10C18H (Knauer, Berlin, Germany). Purified compounds were characterized by matrix-assisted laser desorption ionization time-of-flight mass spectrometry at the Protein and Nucleic Chemistry Facility (University of Cambridge, UK, Supporting information 8). Peptide purity and transition temperature was assessed via polarimetry as previously described [24]. Briefly, peptides were solubilized in 900 μl of 10 mM phosphate buffer with 150 mM NaCl at 2 mg/ml, heated to 70 °C for 10 min and left at 4 °C overnight. Peptide solutions were heated from 8 °C to 80 °C at a ramp-rate of 1 °C/min in an Autopol III polarimeter and the optical rotation was plotted against the temperature (Supporting information 9).

### 4.2 EDC/NHS crosslinking and THP-functionalization of collagen films

Collagen films were prepared as previously described in Grover et al. [16,24]. Briefly, bovine Achilles tendon collagen type I (Sigma, #4387, Gillingham, UK) was suspended in 0.05 M AcOH at a concentration of 0.5% w/v and left to swell overnight at 4 °C. The mixture was homogenized using an Ultra-Turrax VD125 blender for 20 min at 13500 rpm, centrifuged 5 min at 2500 rpm, homogenized 10 min at 13500 rpm and centrifuged 5 min at 2500 rpm. The resulting slurry was left overnight at room temperature before use. 96-well or 12-well plates were coated with respectively 100 μl or 400 μl of slurry and left to dry for three days in a fume hood. Film crosslinking was performed using of a mixture of 1.115 g EDC and 0.276 g NHS per gram of collagen in 75% EtOH for 2h at room temperature (referred to as 100% crosslinking in our previous work). Wells were washed with EtOH 3 × 10 min and deionized water three times for 10 min and left to dry for three days in a fume hood. Photoreactive peptides and GPP10 were diluted to 2.5 μg/ml in PBS (pH 7.4) from a 5 mg/ml stock solution in 0.01 M AcOH. 100 μl on 96-well plates or 400 μl on 12-well plates of peptide solution were added to collagen films and incubated for 30 min in the dark at room temperature. Collagen films were then placed under a long-wavelength UV lamp (Blak-Ray B100AP, 365 nm wavelength) for 5 min and washed with citrate buffer (pH 3) 6 × 2 min and PBS 6 × 2 min.

### 4.3 Cell line generation and culture conditions

H9 hESCs were maintained as colonies as described in Iyer et al. [43].

*Endothelial cells.* ECs were differentiated adapting a protocol from Patsch et al. [44]. 70% confluent H9 hESCs were split as colonies into a 10 cm^2^ cell culture dishes one day prior to protocol initiation. On Day 0, H9-hESC media was aspirated, the dish washed with PBS, and replaced with 6 ml of CDM-PVA containing FGF-2 (20 ng/ml), LY294002 (10 μM) and BMP-4 (10 ng/ml). After 36 h, media was replaced with STEMPRO-34 SFM (Life technologies) containing VEGF-A (200 ng/ml, Peprotech), Forskolin (TOCRIS, 2uM) and Ascorbic Acid (100 mM, Sigma). After 24 h the media was refreshed, and the plate stored for a further 2 to 3 days before magnetic sorting using CD34 conjugated magnetic microbeads (Miltenyi Biotech) according to the manufacturer's instructions. The positively selected cells were then cultured on gelatin coated plates in STEMPRO-34 SFM supplemented with VEGF-A (50 ng/ml) and FGF-2 (8 ng/ml). Media was refreshed every 48 h until seeding, with cells split up to a maximum of 4 passages. Prior to use, 70–90% confluent ECs cultured in 6-well plates were washed with PBS and detached with 300 μl TrypLE for 5 min at 37 °C. TrypLE was quenched with 1 ml of PBS and cells were spun down at 12000 rpm for 4 min. Cells were re-suspended in EGM-2 and 500 μl of cell solution were added on collagen films prepared on 96- or 12-well plates.

*Cardiomyocytes.* H9 hESCs were differentiated to cardiomyocyte using the protocol adapted from Mendjan et al. [45]. Briefly, H9 hESCs were seeded at a density of 10^5^ cells/cm^2^ in a 12 well plate coated with Matrigel (Corning) in CDM-BSA supplemented with ROCK inhibitor (Millipore, 1 μm). Media was changed after 3 h to CMD-BSA, supplemented with FGF-2 (20 ng/ml), Activin-A (50 ng/ml), BMP-4 (10 ng/ml, R&D) and LY294002 (10 μM Tocris). After 42 h media was changed to CDM-BSA supplemented with FGF-2 (8 ng/ml), BMP4 (20 ng/ml), Retinoic Acid (SIMGA, 1 μM), endo-IWRI (1 μM, TOCRIS). Cells were maintained with this media for 4 days with media refreshed every 48 h. Media was changed to CDM-BSA supplemented with FGF-2 (8 ng/ml), BMP4 (20 ng/ml) for an additional 2 days. Media was then changed to CDM-BSA with no cytokines and replaced every 48 h. Spontaneous beating is generally observed 8–10 days after seeding.

### 4.4 RNA extraction, retrotranscription and RT-qPCR

RNA was extracted using GenElute™ Mammalian Total RNA Miniprep Kit (Sigma) according to the manufacturer's instructions. 250 ng of RNA was subsequently retrotranscribed to complementary DNA (cDNA) using Maxima First Strand cDNA Synthesis Kit (Thermo scientific). For CM cardiac genes and collagen-binding integrins, RT-qPCR was performed using Fast SYBR® Green Master Mix on a 7500 Real-Time PCR System using GAPDH as housekeeping gene. All primers for CM cardiac genes (listed in the table below) were designed to span an intron-exon junction. For VWFIII_Nle_-binding proteins, RT-qPCR was performed using Taqman primers and Taqman Gene expression Mastermix reagents (Thermo Fisher) on a 7300 Real-Time PCR System using HPRT1 as housekeeping gene. mRNA relative expression was obtained using the ΔCt method.

**Table:** 

Gene	Fw primer	Rev primer
hGAPDH	AACAGCCTCAAGATCATCAGC	GGATGATGTTCTGGAGAGCC
MYH7	ACTGCCGAGACCGAGTATG	GCGATCCTTGAGGTTGTAGAGC
MYH6	GCCCTTTGACATTCGCACTG	GGTTTCAGCAATGACCTTGCC
TNNI1	CCGGAAGTCGAGAGAAAACCC	TCAATGTCGTATCGCTCCTCA
TNNI3	TTTGACCTTCGAGGCAAGTTT	CCCGGTTTTCCTTCTCGGTG
SCN5A	GAGCTCTGTCACGATTTGAGG	GAAGATGAGGCAGACGAGGA
RYR2	ACAACAGAAGCTATGCTTGGC	GAGGAGTGTTCGATGACCACC
SERCA	ACAATGGCGCTCTCTGTTCT	ATCCTCAGCAAGGACTGGTTT
MYL2	TACGTTCGGGAAATGCTGAC	TTCTCCGTGGGTGATGATG
MYL7	CCGTCTTCCTCACGCTCTT	TGAACTCATCCTTGTTCACCAC

### 4.5 Cell adhesion

96-well plates were coated with crosslinked collagen films functionalized with GFOGER, GLOGEN or VWFIII_Nle_ as described above, or with GPP10 at 10 μg/ml overnight. 100 μl of a suspension of ECs at 5 × 10^5^ cells/ml in sterile PBS containing 5 mM Mg^2+^ were added to wells. Cells were incubated at 37 °C for 20 min and were washed gently with 200 μl of PBS three times. 150 μl of lysis buffer (containing 21 mg/ml disodium citrate, 6 mg/ml citric acid, 0.1% Triton X-100 and 5 mM p-nitrophenyl phosphate) were added for 90 min at room temperature. The reaction was terminated with 50 μl of 2 M sodium hydroxide and absorbance at 405 nm was measured [46].

### 4.6 Cell spreading

500 μl of ECs at 5.10^4^ cells/ml in STEMPRO-34 SFM were added on collagen films prepared on 12-well plates. Cells were incubated at 37 °C with 5% CO_2_ for 45 min, fixed with 3% paraformaldehyde for 15 min and washed three times with 1% BSA in PBS. Cells were permeabilized by adding 500 μl of 0.5% Triton X-100 for 5 min, then stained with 500 μl of Rhodamine-Phalloidin (0.2 U/ml in PBS containing 0.1% BSA) during 45 min. Mean cell size were measured by locating rhodamine fluorescence on 10 randomly selected fields of view acquired on a Leica DM6000 FS fluorescence microscope using identical microscope settings between experiments. Representative fields of view shown in Fig. 2 were acquired on an Olympus FV300 laser-scanning confocal microscope.

### 4.7 Cell proliferation

*For endothelial cells.* 500 μl of ECs at 5.10^4^ cells/ml in STEMPRO-34 SFM were added to collagen films prepared on 12-well plates. Cells were incubated for 24 h at 37 °C with 5% CO2. 20 μl per well of EdU (5-ethynyl-2′-deoxyuridine, Thermo Fisher) at 1 mM was added and cells were incubated for a further 2h at 37 °C with 5% CO_2_ in the dark. Cells were fixed with 3% paraformaldehyde for 15 min, washed three times with 500 μl of 3% BSA in PBS, permeabilized with 1 ml of 0.5% Triton X-100 in PBS for 10 min and washed twice with 500 μl of 3% BSA in PBS. 500 μl per well of Click-iT solution (Thermo Fisher), together with 1:2000 of Hoechst 33342 stain, were added for 30 min in the dark and washed three times with PBS. Percentage of proliferating cells was calculated as the ratio of cell nuclei stained with Alexa-Fluor 488 over nuclei stained with Hoechst 33342 in 10 randomly selected fields of view for each condition acquired on a Leica DM6000 FS fluorescence microscope in three repeats.

*For cardiomyocytes.* Collagen films were washed once with PBS and pre-conditioned with cell culture media for 1 h before cell seeding. Cardiomyocytes were dissociated using TrypLE (Life technologies) and plated at a concentration of 10^5^ cells per film in a 96-well plate, in CDM BSA supplemented with ROCK inhibitor 1μM. 10 μM per well BrdU dissolved in culture medium was added and incubated for 12 h before fixing the cells in 4% paraformaldehyde (PFA). After washing with PBS, cells were incubated in 1.5 M HCl for 30 min (Antigen retrieval step), washed and incubated overnight at 4 °C mouse anti-BrdU (1:50 Becton-Dickinson, Ref: 347580) in 3% BSA/0.5%Triton-X-100 in PBS overnight at 4 °C. Alexa-Fluor 568 Rabbit anti-mouse (Life Technologies Ref: A11061) diluted 1:400 in 3% BSA/0.5%Triton-X-100 in PBS was added and samples were incubated at room temperature for 1 h on a rocker, washed in PBS and incubated with DAPI (Sigma, 1 μg/ml) for 5 min at room temperature. Percentage of proliferating cells was calculated as the ratio of cell nuclei stained with Alexa-Fluor 568 over nuclei stained with DAPI in 5 randomly selected fields of view for each condition acquired on a Zeiss Axiovert inverted microscope in three independent repeats.

### 4.8 LDL uptake

70–90% confluent ECs were starved with EBM with 0.05% FBS for 2h. Cells were then washed with PBS and detached with 300 μl TrypLE for 5 min at 37 °C. TrypLE was cancelled with 1 ml of PBS and cells were spun down at 12000 rpm for 4 min. Cells were re-suspended in STEMPRO-34 SFM at 5.10^4^ cells/ml with 0.5 μl/ml of 1,1′-Dioctadecyl-3,3,3′,3′-Tetramethyl-indo-carbocyanine perchlorate (DiI) conjugated acetylated Low Density Lipoprotein (Ac-LDL) and 500 μl were added on collagen films prepared on 12-well plates. Cells were incubated for 24 h at 37 °C with 5% CO_2_, fixed with 3% paraformaldehyde for 15 min, washed three times with 3% BSA in PBS, permeabilized with 0.5% Triton X-100 in PBS for 10 min and washed twice with 3% BSA in PBS. Cell nuclei were stained with 1:2000 Hoechst 33342 in PBS for 30 min in the dark and washed three times with PBS. Ac-LDL-Dil fluorescence were measured and Hoechst-stained cells were counted in 10 randomly selected fields of view for each condition in four repeats on a Leica DM6000 FS or Zeiss Axio Z1 fluorescence microscope.

### 4.9 Immunocytochemistry and flow cytometry

Collagen films were washed once with PBS and pre-conditioned with cell culture media for 1 h before cell seeding. Cardiomyocytes were dissociated using TrypLE (Life technologies) and plated at a concentration of 10^5^ cells per film in a 96-well plate in CDM BSA supplemented with ROCK inhibitor 1 μM. For co-cultures, endothelial cells were coated alongside at a ratio of endothelial cells to cardiomyocyte of 1:10 in STEMPRO-34 supplemented with VEGF-A (50 ng/ml). Cells were fixed with 4% PFA, permeabilized with 0.1% Triton (Sigma) in PBS for 15 min before blocking with 3% BSA (Sigma) in PBS for 1 h and overnight incubation with primary antibody (diluted accordingly). Cells were then washed in PBS and incubated with the appropriate secondary antibody for 2 h at room temperature, washed and stained with DAPI (Sigma, 1 μg/ml) before imaging on an Axiovert inverted microscope (Zeiss). Primary (I) and secondary (II) antibodies are listed in the table below. Flow cytometry was performed using BD Cytofix/Cytoperm kit (BD biosciences, cat. 554714) according to the manufacturer instruction. Data were acquired on LSRFortessa (BD bioscience) flow cytometer and analysed using FlowJo VX software version 9.9.4.

**Table:** 

Antibody	Supplier	Cat. Number
Goat polyclonal anti Cardiac Troponin T (I)	Abcam	ab64623
Rabbit polyclonal anti CD31 (I)	Novus biologicals	NB100-2284
Mouse monoclonal Anti-BrdU antibody (I)	Sigma	B8434
Mouse monoclonat Anti-Connexin-43 (I)	Millipore	MAB3067
Donkey anti Rabbit Alexa 488 (II)	Life Technologies	R37118
Donkey anti goat Alexa 568 (II)	Life Technologies	A-11057
Donkey anti goat Alexa 488 (II)	Life Technologies	A-11055
Chicken anti-mouse 488 (II)	Life Technologies	A-21200
Donkey anti-mouse 568 (II)	Life Technologies	A10037
Donkey anti-rabbit 647 (II)	Life Technologies	A32795
Mouse anti VECAD-APC	Miltenyi Biotec	130-125-985
Alexa Fluor® 488 Mouse anti-FAK (pS910)	BD bioscience	558544
Mouse anti CD29 -APC	Miltenyi Biotec	130-101-280
Mouse anti CD49a - APC	Miltenyi Biotec	130-101-395
REAfinity anti CD49b - APC	Miltenyi Biotec	130-100-337
Rabbit anti ITGA10	ThermoFisher	PA5-100840
Rabbit anti ITGa11	Biorbyt	184286
Mouse IgG control -FITC	Biolegend	400108
Mouse IgG control - APC	Biolegend	981806

### 4.10 Cell viability

Collagen films were washed once with PBS and pre-conditioned with cell culture media for 1 h before cell seeding. Cardiomyocytes were dissociated using TrypLE (Life technologies) and plated at 10^5^ cells per film in CDM BSA supplemented with ROCK inhibitor 1 μM. For co-cultures, endothelial cells were coated alongside at a ratio of endothelial cells to cardiomyocyte of 1:10 in STEMPRO-34 supplemented with VEGF-A (50 ng/ml). PrestoBlue® Cell Viability Reagent (Thermo Scientific) was added to culture media according to the manufacturer's instructions. Cells were incubated with the dye for 2 h. Media was then sampled and fluorescence at 560 nm analysed using VICTOR Multilabel Plate Reader (PerkinElmer). Media containing PrestoBlue® incubated in empty wells was used as background control.

### 4.11 Fluo-4 AM calcium analysis

Collagen films were washed once with PBS and pre-conditioned with cell culture media for 1 h before cell seeding. Cardiomyocytes were dissociated using TrypLE (Life technologies) and plated at 10^5^ cells per film in CDM BSA supplemented with ROCK inhibitor 1 μM. For co-cultures, endothelial cells were coated alongside at a ratio of endothelial cells to cardiomyocyte of 1:10 in STEMPRO-34 supplemented with VEGF-A (50 ng/ml). Fluo-4 AM (Life technologies) was added to the cell culture media for 30 min at 37 °C. Cells were paced at a frequency of 1.5 Hz using an EDP 30B dual chamber pacer (Biotronix). Videos were recorded on an Axiovert inverted microscope (Zeiss) using a Sony LEGRIA camera. Videos were then analysed with FIJI ImageJ software and using Time Series analyser V3 Plug in. Time to peak and time to 90% decay were calculated using Matlab R2019b.

### 4.12 Statistical analysis

Values shown are mean ± standard error of the mean (s.e.m.), from three or four biologically independent experiments. Mean values were compared using Prism software (GraphPad, San Diego) and 1-way ANOVA or 2-way ANOVA with Dunnett's post-tests, comparing all conditions to crosslinked films with no peptide. Legend **** on figure denotes p < 0.0001, *** denotes p < 0.001, ** denotes p < 0.01, * denotes p < 0.05 and ns denotes non-significant. Comparisons between other pairs of columns were performed using Student's t-test.

## Declaration of competing interest

The authors declare that they have no known competing financial interests or personal relationships that could have appeared to influence the work reported in this paper.

## Data Availability

The raw data required to reproduce these findings are available to download from the supplementary information.
