# Supplementary material for: Modulating hESC-derived cardiomyocyte and endothelial cell function with triple-helical peptides for heart tissue engineering
Source: Biomaterials. 2021 Feb;269:120612. doi: 10.1016/j.biomaterials.2020.120612 (PMC7884910; doi:10.1016/j.biomaterials.2020.120612)
Supplement: Multimedia component 1 [file mmc1.pptx]

## Slide 1
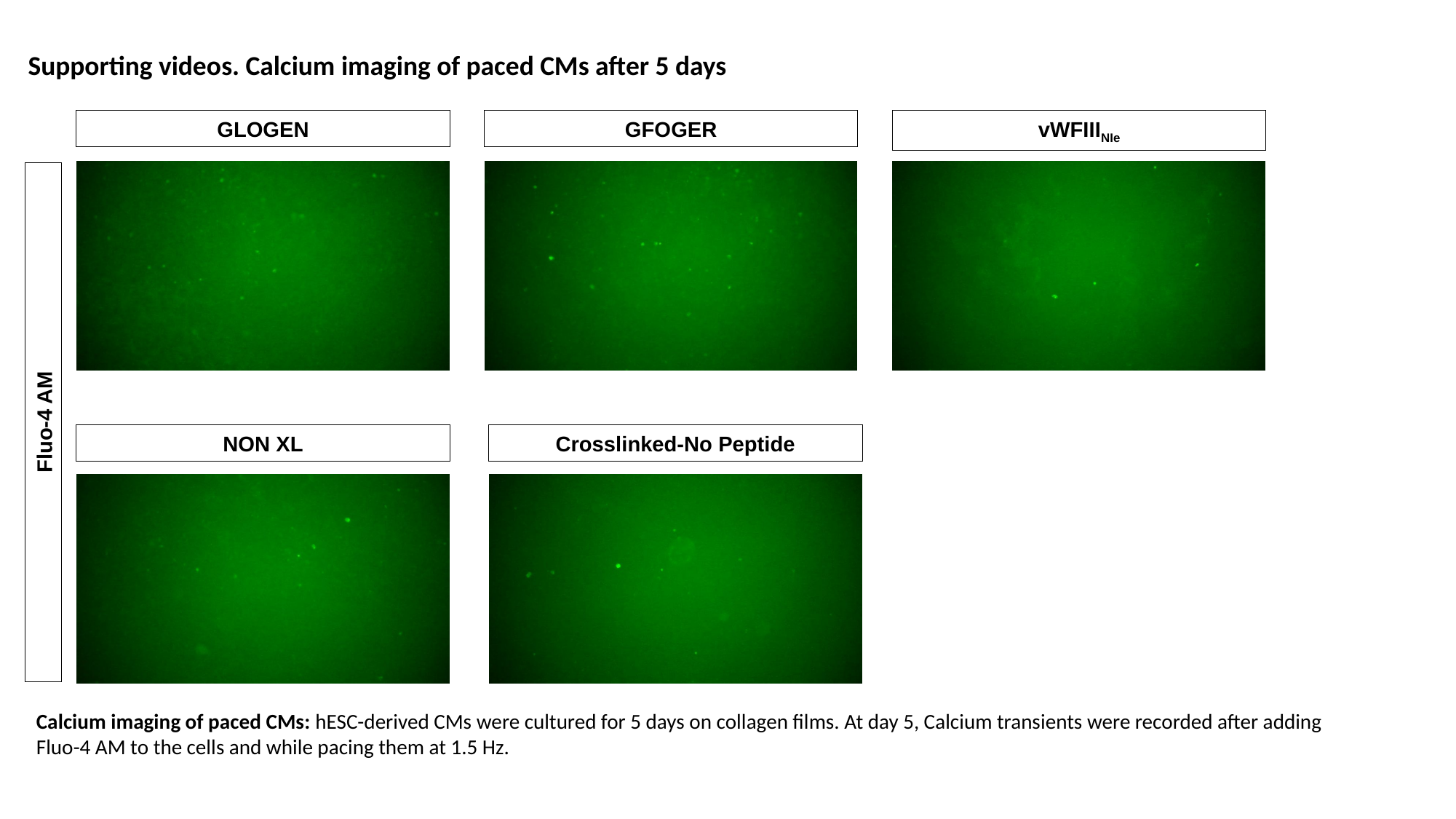

2. SCAFFOLD FUNCTIONALIZATION
Supporting videos. Calcium imaging of paced CMs after 5 days
vWFIIINIe
GLOGEN
GFOGER
Fluo-4 AM
NON XL
Crosslinked-No Peptide
Calcium imaging of paced CMs: hESC-derived CMs were cultured for 5 days on collagen films. At day 5, Calcium transients were recorded after adding Fluo-4 AM to the cells and while pacing them at 1.5 Hz.
