# Supplementary material for: Modulating hESC-derived cardiomyocyte and endothelial cell function with triple-helical peptides for heart tissue engineering
Source: Biomaterials. 2021 Feb;269:120612. doi: 10.1016/j.biomaterials.2020.120612 (PMC7884910; doi:10.1016/j.biomaterials.2020.120612)
Supplement: Multimedia component 3 [file mmc3.docx]

**Modulating hESC-derived cardiomyocyte and endothelial cell function with triple-helical peptides for heart tissue engineering**

Maria Colzani^1*^, Jean-Daniel Malcor^2*^, Emma J. Hunter^2^, Semih Bayraktar^1^, Murray Polkinghorne^1^, Thomas Krieg^3^, Ruth Cameron^4^, Serena Best^4^, Richard W. Farndale^2ⱡ^, Sanjay Sinha^1ⱡ^

1 – Department of Medicine and Wellcome – MRC Cambridge Stem Cell Institute, University of Cambridge, Cambridge, UK

2 – Department of Biochemistry, University of Cambridge, UK

3 – Department of Medicine, University of Cambridge, Cambridge, UK

4 – Department of Materials Science and Metallurgy, University of Cambridge, UK

*These authors contributed equally

^ⱡ^ These authors contributed equally

**Supporting information.**

**Supporting information 1.**


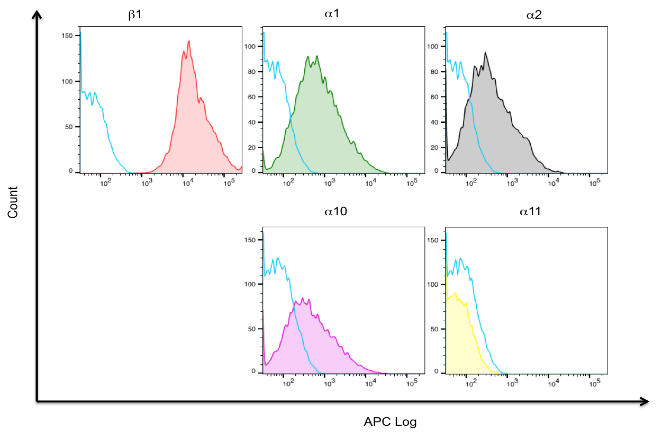

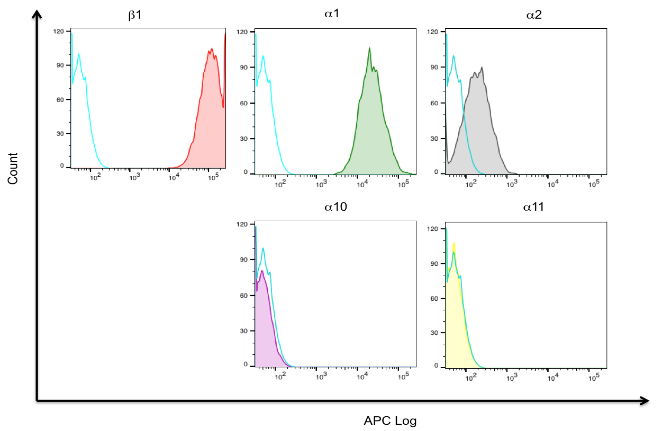


**A**

**B**

**C**

**D**

**Collagen-binding integrin protein expression. A-B.** Representative histograms showing expression of integrin β1 (red), α1 (green), α2 (grey), α10 (magenta) and α11 (yellow) against isotype control (blue) for cardiomyocytes (A) and endothelial cells (B). **C-D.** Mean fluorescence intensity (MFI) for the α subunits was normalized to the MFI of the β1 subunit and plotted for cardiomyocytes (C) and endothelial cells (D). Data represent the average of three independent experiments ± standard deviation.

**Supporting information 2.**

**Cross-reactivity between GFOGER and GLOGEN**. Adhesion studies were performed on of C2C12 cells transfected with the alpha subunit of collagen-binding integrins (α1, α2, α10 or α11) on tissue culture plates coated overnight with 10 μg/ml of GFOGER, GLOGEN or the control peptide GPP10. Results show a relatively higher binding to α1β1 and α10β1 with GLOGEN, and a relatively higher binding to α2β1 and α11β1 with GFOGER. Nevertheless, GLOGEN also binds to α2β1 and α11β1 while GFOGER also binds to α1β1 and α10β1. Columns represent the mean of three independent experiment ± s.e.m (one-way ANOVA, p < 0.001).

**Supporting information 3.**


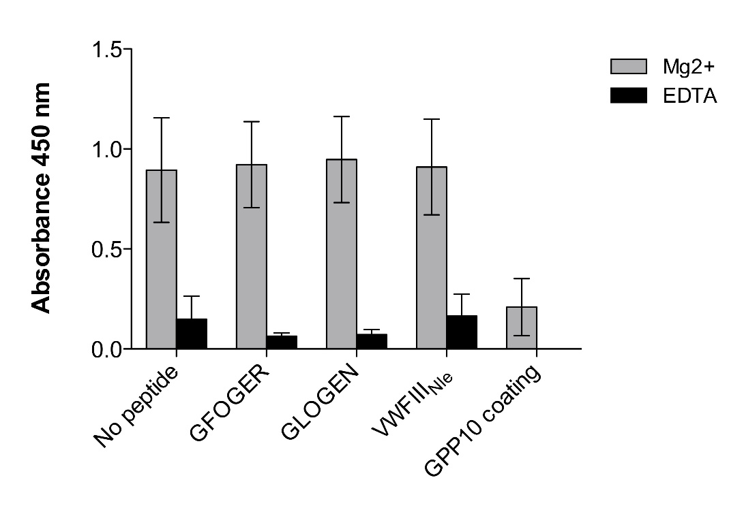


**Endothelial cell adhesion to collagen films.** ECs were incubated at 37°C for 20 min in the presence of either Mg^2+^ or EDTA, washed gently with PBS and lysed with p-nitrophenol containing buffer. Adhesion was quantified by measuring the absorbance of the p-nitrophenol product at 405 nm as described in [1]. Columns represent the mean of three independent experiment ± s.e.m.

**Supporting information 4.**


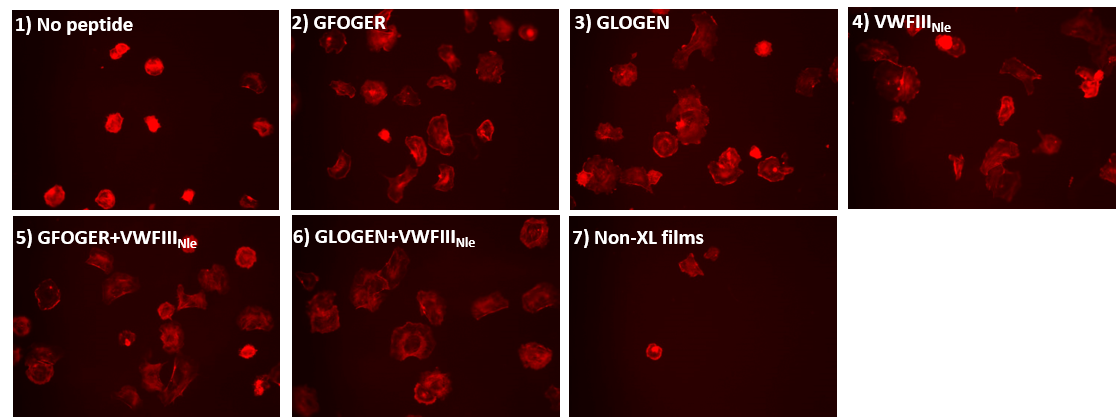


**Endothelial cell spreading.** Representative field of view of EC spreading after 45 min at 37°C with 5% CO_2_. hESC-derived ECs were fixed and stained with Rhodamine-Phalloidin.

**Supporting information 5.**


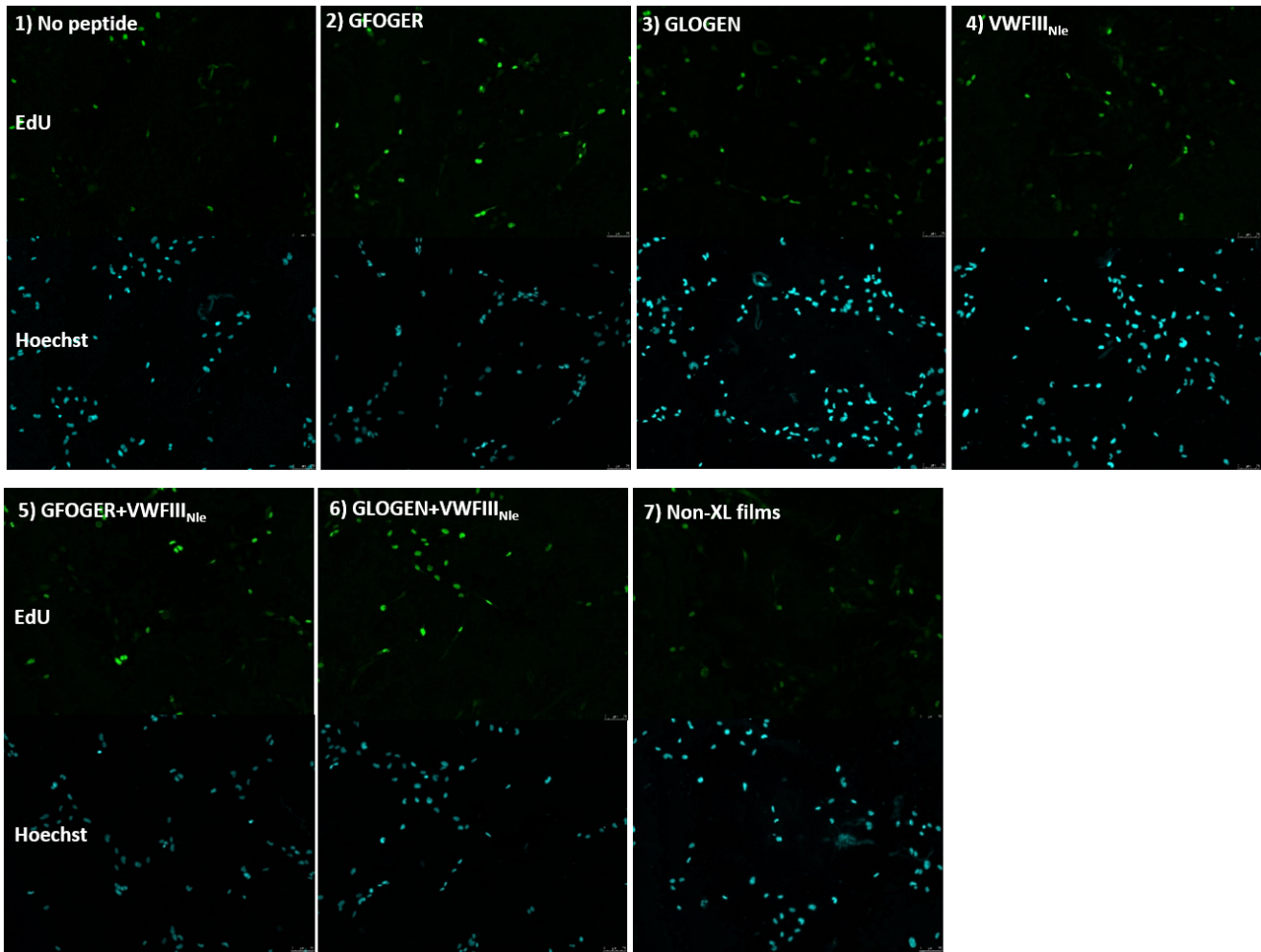


**Endothelial cell EdU staining.** Representative fields of view of ECs cultured for 24h at 37°C with 5% CO_2_. EdU was introduced in the media and cells were left for 2h further. Cells were fixed, and nuclei were stained with Hoechst 33342 and internalized EdU with Alexa-488.

**Supporting information 6.**


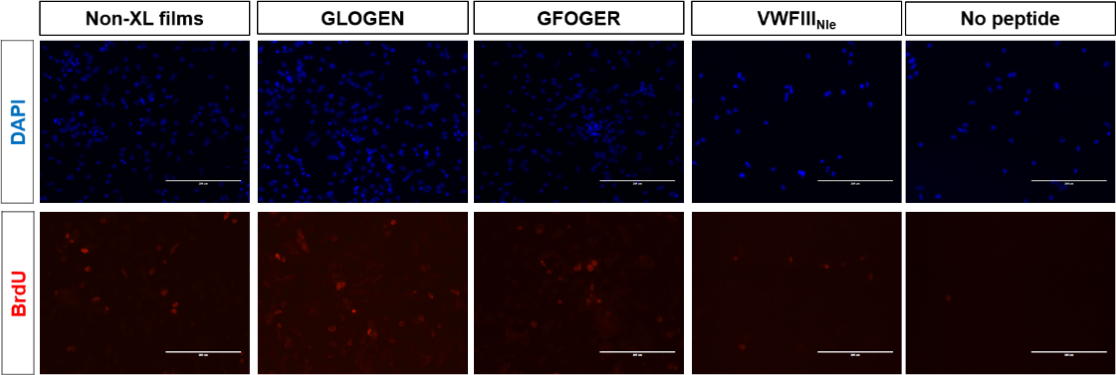


**Cardiomyocytes BrdU staining.** Representative field of view of CMs cultured for 3 days at 37°C with 5% CO_2_. BrdU was introduced in the media and cells were left for 12h further. Cells were fixed and, nuclei were stained with DAPI and internalized BrdU with Alexa-568. Scale bar 100 µm.

**Supporting information 7.**


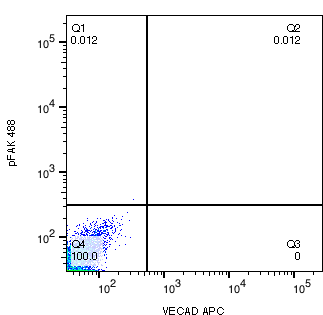

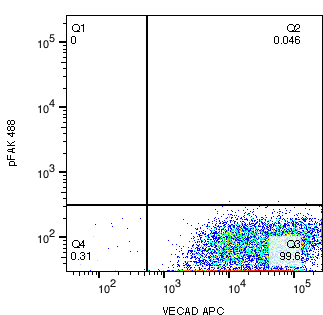


**CM only VECAD staining**

**EC only VECAD staining**


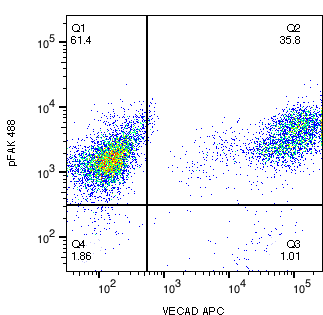


**GLOGEN**


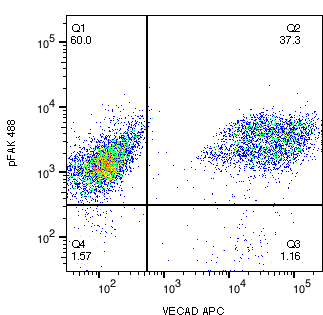

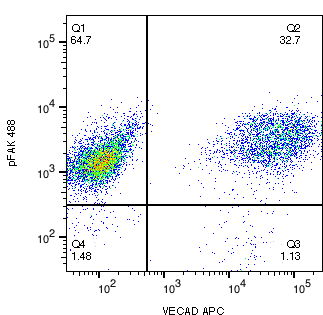


**GFOGER**

**VWFIII_NIe_**


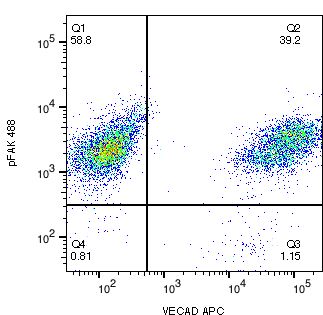


**GLOGEN + VWFIII_NIe_**

**GFOGER + VWFIII_NIe_**


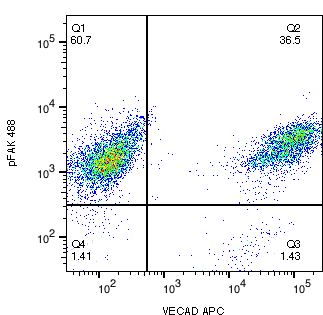

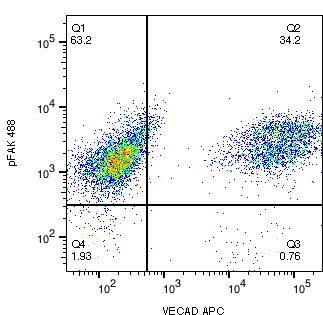


**Non-XL**

p-FAK

VECAD

**A**


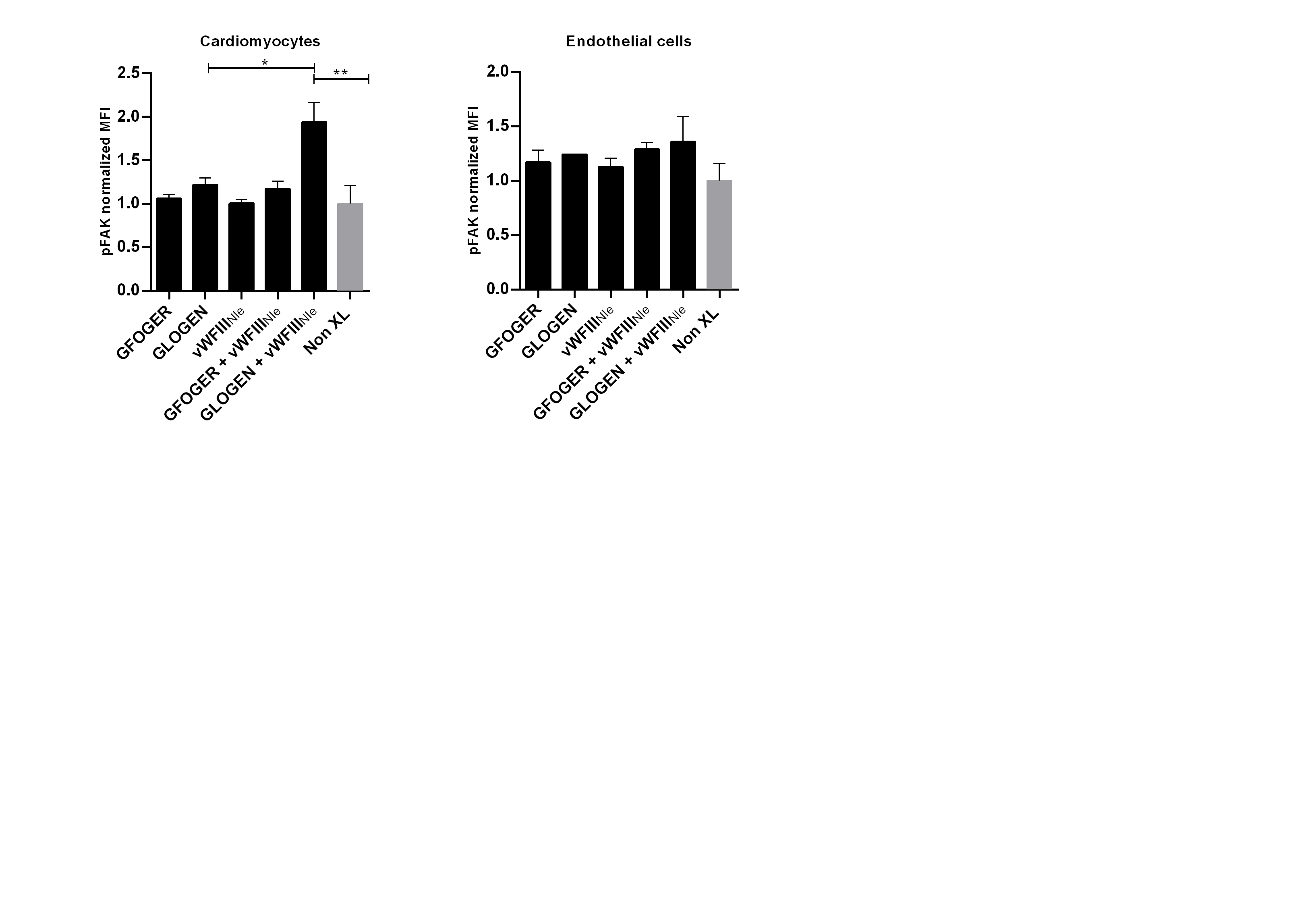


**C**

**B**

**Phosphorylation of FAK in EC-CM co-cultures.** p-FAK levels were measured in EC:CM co-culture by flow cytometry. **A**. Representative flow cytometry dot plots. To distinguish between EC and CM, in addition to p-FAK, cells were stained for VE-Cadherin (VECAD), an endothelial cell specific marker, as shown in top dot plots (CM only and EC only VECAD staining). Mean fluorescence intensity (MFI) was normalized to the non-crosslinked group and plotted for CM and EC (**B** and **C** respectively). Data represents the average of three independent experiment ± standard deviation (one-way ANOVA, * p < 0.05, **p < 0.01.

**Supporting information 8.**


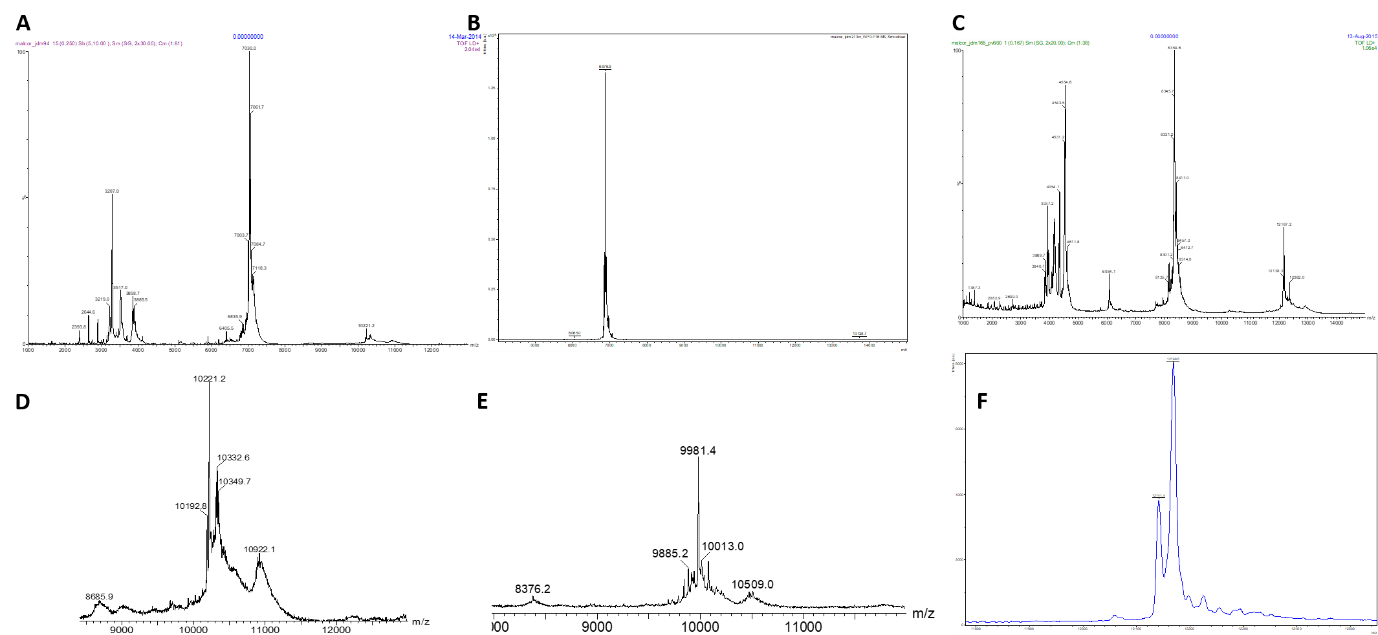


**Matrix-assisted laser desorption ionization time-of-flight (MALDI) mass spectra of THPs.**

**A)** MALDI spectra of Diaz-ES-((GPP)5GFOGER(GPP)5)3 (**GFOGER**) in the range of 1000 m/z to 13000 m/z; peaks at 7030.0 m/z are attributed to species with only two covalently linked peptidic strands due to in source fragmentation**.**

**B)** MALDI spectra of Diaz-ES-((GPP)5GLOGEN(GPP)5)3 (**GLOGEN**) in the range of 4000 m/z to 13000 m/z; peaks at 6876.9 m/z are attributed to species with only two covalently linked peptidic strands due to in source fragmentation.

**C)** MALDI spectra of Diaz-ES-(Ahx(GPP)5GPRGQOGVNleGFO(GPP)5)3 (**VWFIII_Nle_**) in the range of 1000 m/z to 13000 m/z; peaks at 8331.2 m/z are attributed to species with only two covalently linked peptidic strands due to in source fragmentation**.**

**D**) MALDI spectra of Diaz-ES-((GPP)5GFOGER(GPP)5)3 (**GFOGER**) in the range of 8000 m/z to 13000 m/z; peak at 10192.8 m/z is attributed to N_2_ loss from the Diazirine group due to in source fragmentation.

**E)** MALDI spectra of Diaz-ES-((GPP)5GLOGEN(GPP)5)3 (**GLOGEN**) in the range of 8000 m/z to 13000 m/z

**F)** MALDI spectra of Diaz-ES-(Ahx(GPP)5GPRGQOGVNleGFO(GPP)5)3 (**VWFIII_Nle_**) in the range of 8000 m/z to 13000 m/z; peak at 12141.4 m/z is attributed to N2 loss from the Diazirine group due to in source fragmentation

**Supporting information 9.**


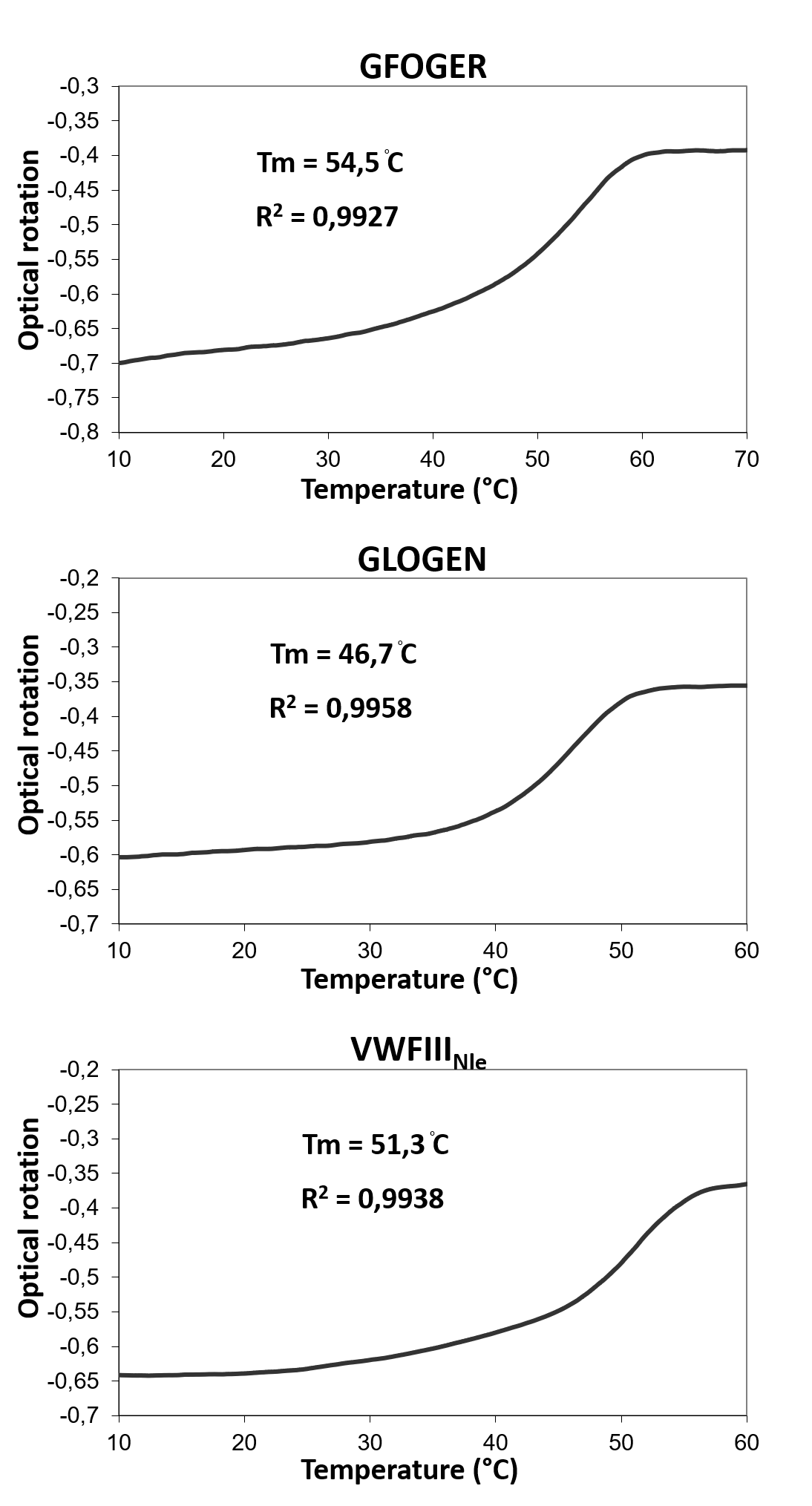


**THP melting temperature assessed by polarimetry.** The optical rotation of THP solutions measured by polarimetry (in degrees) were plotted against the temperature. Previous work carried out in our lab demonstrated that a homogenous triple helix population yields a sigmoidal curve due to the unfolding of the triple helical structure when temperature increases [2]. The transition temperature was measured from these sigmoidal curves (54.5֯ C, 46.7 ֯C and 51.3 ֯C for GFOGER, GLOGEN and VWFIII_Nle_ respectively), non-linear regression was performed and R^2^ was calculated (0.9927, 0.9958 and 0.9938 for GFOGER, GLOGEN and VWFIII_Nle_ respectively).

**References**

[1] D.J. Onley, C.G. Knight, D.S. Tuckwell, M.J. Barnes, R.W. Farndale, Micromolar Ca2+ concentrations are essential for Mg2+-dependent binding of collagen by the integrin alpha 2beta 1 in human platelets, J. Biol. Chem. 275(32) (2000) 24560-24564.

[2] D.A. Slatter, D.G. Bihan, R.W. Farndale, The effect of purity upon the triple-helical stability of collagenous peptides, Biomaterials. 32 (2011) 6621–6632.
